# Combination of linkage and association mapping with genomic prediction to infer QTL regions associated with gray leaf spot and northern corn leaf blight resistance in tropical maize

**DOI:** 10.3389/fgene.2023.1282673

**Published:** 2023-11-07

**Authors:** Dennis O. Omondi, Mathews M. Dida, Dave K. Berger, Yoseph Beyene, David L. Nsibo, Collins Juma, Suresh L. Mahabaleswara, Manje Gowda

**Affiliations:** ^1^ Department of Crops and Soil Sciences, School of Agriculture, Food Security and Environmental Sciences, Maseno University, Kisumu, Kenya; ^2^ Crop Science Division Bayer East Africa Limited, Nairobi, Kenya; ^3^ Department of Plant and Soil Sciences, Forestry and Agricultural Biotechnology Institute (FABI), University of Pretoria, Pretoria, South Africa; ^4^ The Global Maize Program, International Maize and Wheat Improvement Center (CIMMYT), Nairobi, Kenya

**Keywords:** maize, gray leaf spot, northern corn leaf blight, quantitative trait loci, association mapping, genome-wide association study

## Abstract

Among the diseases threatening maize production in Africa are gray leaf spot (GLS) caused by *Cercospora zeina* and northern corn leaf blight (NCLB) caused by *Exserohilum turcicum*. The two pathogens, which have high genetic diversity, reduce the photosynthesizing ability of susceptible genotypes and, hence, reduce the grain yield. To identify population-based quantitative trait loci (QTLs) for GLS and NCLB resistance, a biparental population of 230 lines derived from the tropical maize parents CML511 and CML546 and an association mapping panel of 239 tropical and sub-tropical inbred lines were phenotyped across multi-environments in western Kenya. Based on 1,264 high-quality polymorphic single-nucleotide polymorphisms (SNPs) in the biparental population, we identified 10 and 18 QTLs, which explained 64.2% and 64.9% of the total phenotypic variance for GLS and NCLB resistance, respectively. A major QTL for GLS, *qGLS1_186* accounted for 15.2% of the phenotypic variance, while *qNCLB3_50* explained the most phenotypic variance at 8.8% for NCLB resistance. Association mapping with 230,743 markers revealed 11 and 16 SNPs significantly associated with GLS and NCLB resistance, respectively. Several of the SNPs detected in the association panel were co-localized with QTLs identified in the biparental population, suggesting some consistent genomic regions across genetic backgrounds. These would be more relevant to use in field breeding to improve resistance to both diseases. Genomic prediction models trained on the biparental population data yielded average prediction accuracies of 0.66–0.75 for the disease traits when validated in the same population. Applying these prediction models to the association panel produced accuracies of 0.49 and 0.75 for GLS and NCLB, respectively. This research conducted in maize fields relevant to farmers in western Kenya has combined linkage and association mapping to identify new QTLs and confirm previous QTLs for GLS and NCLB resistance. Overall, our findings imply that genetic gain can be improved in maize breeding for resistance to multiple diseases including GLS and NCLB by using genomic selection.

## 1 Introduction

Despite its importance, maize production in Kenya is still low with an estimated average production of 1.8 t/ha^-1^ among smallholder farmers when compared to the country’s potential production yield of 6 t/ha^-1^ (Munialo et al., 2019). This is partly due to the threat of highly destructive and virulent fungal pathogens limiting crop production (Beyene et al., 2019). In this context, northern corn leaf blight (NCLB), also known as northern leaf blight (NLB) or Turcicum leaf blight (TLB), caused by *Exserohilum turcicum* (Pass.) (Leonard and Suggs, 1974), and gray leaf spot (GLS) caused by *Cercospora zeina* Crous & U Braun (Crous et al., 2006) on the African continent (Nsibo et al., 2019; Nsibo et al., 2021) are the most lethal and economically significant foliar diseases of maize (Beyene et al., 2019; Sserumaga et al., 2020).

The two diseases reduce the photosynthetic potential of a plant and eventually decrease the grain yield (Saito et al., 2018). GLS is characterized by tan-to-gray rectangular lesions that are limited within the leaf veins (Korsman et al., 2012). It is associated with yield losses of approximately 30%–50%, particularly when using susceptible lines (Kinyua et al., 2010). On the other hand, NCLB is characterized by long elliptical cigar-shaped lesions on leaves that are gray–green (Welz, 1998). NCLB or TLB has been reported to cause yield reductions of 36%–72% in susceptible maize genotypes (Berger et al., 2020). The high genetic diversity reported for *C. zeina* and *E. turcicum* in Kenya (Borchardt et al., 1998; Nsibo et al., 2021) could lead to recombining pathogen populations, hence posing a greater risk to the vulnerable susceptible lines (McDonald and Linde, 2002). Therefore, there is a need to continuously discover new sources of resistance.

The doubled haploid (DH) technology offers the fastest alternative to achieve 100% homozygosity (attained in two generations) which is essential for a mapping population and population improvement (Prasanna et al., 2021). To complement the DH technology, genotyping by sequencing platforms such as Diversity Arrays Technology (DArT) offers a high-throughput platform for genotyping single-nucleotide polymorphism (SNP) markers (Kilian et al., 2012; Sansaloni et al., 2020). The DArTseq platform is purposefully a powerful tool for genome-wide discovery of SNP markers without prior sequence information (Wenzl et al., 2004). In addition, it generates high-density linkage maps, and it is also cost-competitive (Jaccoud et al., 2001; Sánchez-Sevilla et al., 2015).

Complex traits such as GLS and NCLB resistance are controlled by polygenic genes with minor effects that are distributed throughout the genome (Welz and Geiger, 2000; Wisser et al., 2006; Poland et al., 2011; Van Inghelandt et al., 2012; Chen et al., 2015; Ding et al., 2015). Mapping of the quantitative trait loci (QTLs) based on linkage analysis is a powerful tool for identifying the genomic regions associated with the traits of interest. Previous QTL studies have mapped QTL for resistance to GLS and NCLB on all 10 maize chromosomes (Berger et al., 2014; Chen et al., 2015). A number of these QTLs have been fine-mapped with others cloned and the molecular mechanisms underlying such QTL characterized. Additionally, QTL mapping offers the advantage of mapping as early as at the F_2_ populations; however, this is characterized by the limited number of recombination events captured and sizeable confidence interval (Challa and Neelapu, 2018; Rashid et al., 2020).

Genome-wide association studies (GWAS) attempt to overcome the drawbacks of QTL mapping as they utilize age-old recombination events in a large array of unrelated individuals leading to high-speed decay of linkage disequilibrium (Xiao et al., 2017; Kolkman et al., 2020). GWAS studies dig into the entire genome of different varieties (considering the SNPs present in the genotypic data) to establish the link between genotypic variations and the corresponding trait (Challa and Neelapu, 2018). Kibe et al. (2020a) combined the use of linkage mapping and GWAS to detect the significant SNPs and QTL conditioning resistance to GLS in an Improved Maize for African Soils (IMAS) diversity panel and a set of DH populations in Kenya. Several putative candidate genes involved in the transportation channel were identified to have a role in plant defense. In the present study, we attempted to validate some of the GLS resistance QTLs reported in the study by Kibe et al. (2020a) by using a common tropical parent CML511.

Genomic prediction (GP) is another promising genomic tool that has been applied successfully in plant breeding programs (Crossa et al., 2017). Previous studies indicated the potential of GP to increase genetic gain and reduce the time taken in breeding programs significantly (Beyene et al., 2019; Kibe et al., 2020a; Kibe et al., 2020b). In contrast to genetic mapping which identifies significant marker–trait associations, GP uses all markers available to estimate their effects, thus providing a powerful approach to account for any effects that might have been missed by either genetic or association mapping (Beyene et al., 2019). GP exhibits a close relationship to GWAS owing to the large genomic and phenotypic datasets used by the methods (Beyene et al., 2021).

However, this does not mean the complete withdrawal of genetic mapping but rather the incorporation of the two in genetic studies as complementary approaches since each provides considerable advantages. With this background, the objectives of this study were as follows: (1) to phenotypically characterize an elite tropical DH population and 240 tropical and sub-tropical maize inbred lines panel for their responses to GLS and NCLB, including correlation with other agronomic traits; (2) to identify population-based common QTL regions and significant SNPs using GWAS and linkage mapping; and (3) to assess the usefulness of GP in breeding for GLS and NCLB resistance in tropical maize.

## 2 Materials and methods

### 2.1 Study sites and genetic material

This study used (i) a biparental DH population derived from the tropical×tropical germplasm CML511×CML546 inbred lines and (ii) an association panel made up of a collection of 239 tropical and sub-tropical maize inbred lines with early and intermediate maturity in Eastern Africa, representing some of the genetic diversity (for low N, drought, and biotic stresses, Beyene et al., 2021; Prasanna et al., 2021). The association panel was evaluated in three locations in western Kenya, at Kitale (1.0191° N and 35.0023° E, 1900 masl); Shikutsa (0°16′57.83″N and 34°45′6.71″E, 1561 masl); and Kakamega (0°17′3.19″N and 34°45′8.24″E, 1535 masl). The biparental population was evaluated across different ecologies in western Kenya; Maseno University field demonstration site in 2018 (0°00′18.2″S and 34°35′43.5″E, 1500masl), Maseno 2019 (0°00′07.0″S and 34°35′41.9″E, 1503 masl), and farmer’s field in Kabianga 2018 (0°25′24.1″S and 35°07′31.7″E, 1780 masl).

### 2.2 Experimental design

Two hundred and thirty (230) entries (228 DH lines and two parents) of the biparental population were planted in a 5 × 46 alpha lattice design, randomized, and replicated three times at each site by using the CIMMYT’s field book (Vivek et al., 2007). The association panel was also planted in 5 × 48 alpha lattice design, randomized, and replicated two times in each of three environments. Experimental plots consisted of 3 m long single rows with the rows spaced at 0.6 m apart. Adjacent plots were planted 0.75 m apart with an alley of 1.2 m at the end of each plot. Each plot was planted with 13 hills, with two seeds getting planted per hill. Thinning was later conducted to one plant per hill. Border rows of susceptible genotypes were also planted to act as spreaders of the pathogen. The experimental plots were managed using standard agronomic practices.

### 2.3 Phenotypic evaluation and data collection

GLS and NCLB disease severity (DS) were scored on a per-row basis using an ordinal 1–9 scale adapted from the work of Berger et al. (2014) for GLS and Berger et al. (2020) for NCLB. For DH population, DS ratings for GLS and NCLB were taken once per week for at least 5 weeks starting on average at 15 days after flowering (R2; reproductive stage two). All the data were collected using the CIMMYT’s field book (Vivek et al., 2007). The DS scale was used as follows: score 1 for no GLS or NCLB lesions visible on the entire plant, 2 indicated close inspection of each leaf is necessary to find lesions, 3 indicated lesions are more easily seen but are majorly restricted to leaves lying below the ears, 4 indicated individual lesions are just becoming visible on the ear leaf and the leaves above the ears, 5 indicated lesions are more visible on the leaves above the ears, with the infections capturing <10% of the top leaves, 6 indicated lesions are more easily seen on the leaves above the ear leaf with infections covering >10% of the leaf area, 7 for GLS and NCLB lesions dominating the leaf area on all the leaves with 50% of the maize leaf surface diseased, 8 for GLS and NCLB lesions prevalent on all the leaves of the maize plant with 80% of the maize leaf surface diseased, and 9 for GLS and NCLB lesions prevalent on all the leaves of the maize plant with the maize plant exhibiting a gray appearance with >80% of the maize leaf area diseased. For both GLS and NCLB, the DS scores over five intervals were used to calculate the area under the disease progress curve (AUDPC, Shaner, 1977). For the GWAS panel, both DS data were recorded at the dough stage of the plants. For DH population, data were also collected on days to anthesis (AD**,** the number of days from planting to when 50% of the plants in a plot were shedding pollen), days to silking (SD, the number of days from planting to when 50% of maize crops in a plot were showing silk), plant height (PH, cm), and ear height (EH, cm). Maize development stages were recorded using the scale of Purdue University (http://extension.entm.purdue.edu/fieldcropsipm/corn-stages.php).

### 2.4 Statistical analysis of the phenotypic data

Multi-environment trial analysis with R for windows (META-R) version 6.0 (Alvarado et al., 2015) was used to obtain the best linear unbiased estimations (BLUEs) and best linear unbiased predictions (BLUPs). In addition to BLUEs and BLUPs, META-R was also used to compute the genetic correlations among all the variables and among environments, least significant difference (LSD), grand mean, variance components, coefficients of variation (CV), and broad-sense heritability for all the variables. Analysis of the phenotypic data for both biparental population and association panel was conducted both within and across environments.

The BLUEs and the BLUPs were calculated for DS of GLS and NCLB, PH, EH, AD, SD, and the AUDPC which were the response variables. The columns in the input files were selected to be the factor names with the environment, replicate, block, and genotype as the independent variables. For analysis across environments using a lattice design, the following linear mixed model was used.

Y_*ijkl*_ = *µ* +Env_*i*_ + Rep_*j*_(Env_*i*_) +Block_*k*_(Env_*i*_ Rep_*j*_) +Gen_*l*_ + Env_*i*_ ×Gen_*l*_ + ε_*ijkl*_.

From the aforementioned equation, Y_*ijkl*_ represents the performance of the trait of interest, *µ* corresponds to the all-inclusive mean, Env_*i*_ represents the effect of the *i*th environment, Rep_*j*_(Env_*i*_) represents the effect of the *jth* replication within the *ith* environment, Block_*k*_(Env_*i*_ Rep_*j*_) represents the effect of the *kth* incomplete block within the *j*th replication in the *ith* environment, Gen_*l*_ represents the effect of the *lth* genotype, Env_*i*_ ×Gen_*l*_ represents the environment by genotype interaction, and ε_*ijk*_ is the error variance. When calculating the BLUEs, genotypes and covariates were considered fixed effects of the model while other terms were included as random effects of the model. The covariate was considered as fixed effect of the model while all other terms were included in the random effects of the model to estimate the BLUPs. Heritability for the different traits was calculated as the ratio of the estimated genotypic variance to the estimated phenotypic variance (Knapp et al., 1985).

### 2.5 Genotyping and QTL mapping

The CML511×CML546 DH and parental lines were grown in a greenhouse. Maize leaf tissue samples were collected from young, healthy seedlings at the V3 stage (3–4 weeks old), stored at −80°C, and later freeze-dried for 72 h. High-quality genomic DNA was isolated from freeze-dried tissues using the standard CIMMYT laboratory protocol (CIMMYT, 2005). The DH lines were genotyped using DArTSeq in Canberra, Australia. Approximately 15,000 SNPs were used for further quality checks (Murithi et al., 2021). Trait Analysis by aSSociation Evolution and Linkage (TaSSEL) (Bradbury et al., 2007) was used to summarize genotype data by site, determine the allele frequencies, and implement quality screening. All SNP variants that were monomorphic between the two parents, had heterozygosity of >0.05 and a minor allele frequency of <0.05, were filtered, and 1,264 high-quality SNPs were retained for QTL mapping.

Redundant markers were removed using the BIN tool in QTL IciMapping v.4.2 (Meng et al., 2015). In the parameter setting window for general information, eight functionalities were used to define the mapping population. In the indicator row, 1 was selected to denote QTL mapping in actual populations and 3 as the population type as this study used a DH population. Kosambi was set as the mapping function, marker position as the marker space type, 10 as the number of chromosomes, 230 as the size of the mapping population, and 6 as the number of traits.

The number of markers in each chromosome was specified in the chromosome information part. The scores for all the DArTseq markers were transformed into genotype codes following the scores of the parents (2 denoted the marker type of the first parent, 0 denoted the second parent, 1 for the F_1_ marker type, and −1 for the missing markers) (Meng et al., 2015). The genetic linkage maps were constructed using the MAP functionality of QTL IciMapping v.4.2 (http://www.isbreeding.net). Three steps were followed in linkage map construction: grouping, ordering, and rippling. The logarithm of odds score was set at 3.0 for grouping. Ordering was performed using the ordering instruction with the nnTwo Opt algorithm. The sum of adjacent recombination frequencies (SARF) as the criterion and window size of 5 as the amplitude were used to ripple the marker sequence and to fine-tune the chromosome orders. All the outputting functionalities were checked, and the map was drawn using the MAP functionality (Meng et al., 2015).

In the phenotypic data, the BLUPs for the different traits were used as the input files for QTL identification across environments (Littell et al., 2007). The input file was loaded onto the project of IciMapping v.4.2 (Meng et al., 2015). In the parameter setting window ICIM-ADD, other parameters, such as step in scanning represented by cM and stepwise regression of phenotype on marker variables, were defined. An LOD threshold of 3.0 and 1000 permutations at α = 0.01 were set to declare the significant QTL. The percentage of total phenotypic variance explained by an individual QTL was determined using stepwise regression. To ascertain the actual locations of the QTL for all the traits on the chromosomes, the physical position of the identified QTL was assigned based on the known physical position of the linked markers and also available at the maize genetics and genomics database (http://www.maizegdb.org/data_center/map), as described by Berger et al. (2014). The individual QTLs were assigned names according to the QTL, trait name, chromosome, and marker position, as described in the work of Kibe et al. (2020a).

### 2.6 Genotyping and association mapping

The DNA of all 239 inbred lines of the association panel was extracted from seedlings at the 3–4 leaf stage and genotyped using the genotype-by-sequencing (GBS) platform at the Institute for Genomic Diversity, Cornell University, Ithaca, United States, using high-density markers, as per the procedure described by Elshire et al. (2011). SNP calling and imputation were conducted at Cornell University. For SNP calling, raw data in a FASTQ file together with the barcode information and Tags On Physical Map (TOPM) data, which had SNP position information, were used. We used TOPM data from AllZeaGBSv2.7 downloaded from Panzea (https://www.panzea.org/), which contained information for 955,690 SNPs mapped with B73 AGPv2 coordinates. SNP calling was then performed using the TASSEL-GBS pipeline (Glaubitz et al., 2014; Wang et al., 2020). Using TASSEL ver5.2 (Bradbury et al., 2007), SNPs with a heterozygosity of >5%, MAF of >0.05, and minimum count of 90% were excluded by filtering from raw GBS datasets, and 230,743 high-quality SNPs were retained for further analysis in the association panel. To explore the population structure and the ultimate number of subpopulations, principal component analysis (PCA) as described by Price et al. (2006) was conducted in TASSEL using SNPs across all panels. The first three principal components were instrumental to visualize the existing population stratification within the association panel, and this was clearly displayed in a 3D plot. The PCA plots of the association panel were computed using 230,743 SNPs; we then plotted the variance (y-axis) against the principal components (x-axis) to estimate the number of clusters within the population (Sanchez et al., 2018). The data point at which the increase in the number of principal components did not result in an increase in variance (leveling off) indicated the number of subgroups within the panel. To estimate the amount of genetic relatedness among individuals, a kinship matrix was explored.

GWAS was performed with different models to compare and choose the appropriate model with relatively less false positives. To detect marker–trait associations, GWAS was performed using the following models: (1) mixed linear models (MLMs; PCA + K + G) that incorporated PCA, kinship (K), and genotypic data as covariates; (2) the general linear model (GLM; PCA + G) which incorporated the genotype data (G) and the PCA (Q) that both acted as fixed effects to correct for the population structure; and (3) Fixed and random model Circulating Probability Unification (FarmCPU), in which the kinship (random) and the three-component analysis (fixed) were identified as covariates (Lipka et al., 2012). Single-locus GWAS models such as the GLM are characterized by high false positive rates, as a complementary model, and the MLM utilizes the Bonferroni correction to overcome the challenge of false positive rates and identify the loci of interest (Khan et al., 2021). The software TASSEL (Bradbury et al., 2007) was instrumental to run the GLM + PCA and MLM. The − log 10 *p* values for all the analyzed SNPs for both GLS-DS and NCLB-DS were used to construct the Manhattan plots. Q–Q plots were plotted from the estimated -log_10_ (*p*) from the association panel for GLS-DS and NCLB_DS traits. Analysis of the association panel was conducted in TASSEL based on 230,743 filtered SNPs. The R package ‘FarmCPU’ with the Genome Association and Prediction Integrated Tool (GAPIT) was used for GWAS analysis (Tang et al., 2016). The false discovery rate (FDR) was calculated for significant associations using the Benjamini and Hochberg (1995) correction method, with 8 × 10^−5^ as the threshold. To summarize GWAS results per chromosome, Manhattan scatter plots were generated by plotting genomic positions of the SNPs against their negative log base 10 of the *p*-values obtained from the GWAS model, with the F-test for the null hypothesis on the y-axis.

SNPs detected in the association panel were examined as polymorphisms in linkage disequilibrium with putative candidate genes from the B73 reference gene set (https://phytozome-next.jgi.doe.gov/jbrowse/index.html?data; *Zea mays* Zm-B73-REFERENCE-NAM-5.0.55) (Goodstein et al., 2012). Putative candidate genes were selected by delving into the information from Gene Ontology, Kyoto Encyclopedia of Genes and Genomes (KEGG), and protein families (Pfam) (Ashburner et al., 2000; Kanehisa and Goto, 2000; Bateman et al., 2004).

Genomic prediction was carried out with ridge regression BLUP (Zhao et al., 2012) within a biparental DH population for GLS, NCLB, and agronomic traits at five-fold cross-validation. The BLUEs across environments were used for the analysis. The same set of 1,264 high-quality uniformly distributed SNPs with no missing values and MAF>0.05 were used. For the maize association panel, quality screening criteria of SNPs with MAF >0.10 and no missing values were applied, and finally, 8,365 SNPs from the 230,743 SNPs were retained for the analyses. The prediction was ‘within population’, where training and validation sets were derived from within the biparental population. For each trait, 100 iterations were carried out for the sampling of the training and validation sets. The prediction accuracy was calculated as the correlation between the observed phenotypes and genomic estimated breeding values (GEBVs) divided by the square root of heritability (Dekkers, 2007).

## 3 Results

### 3.1 Phenotypic data

**FIGURE 1:**
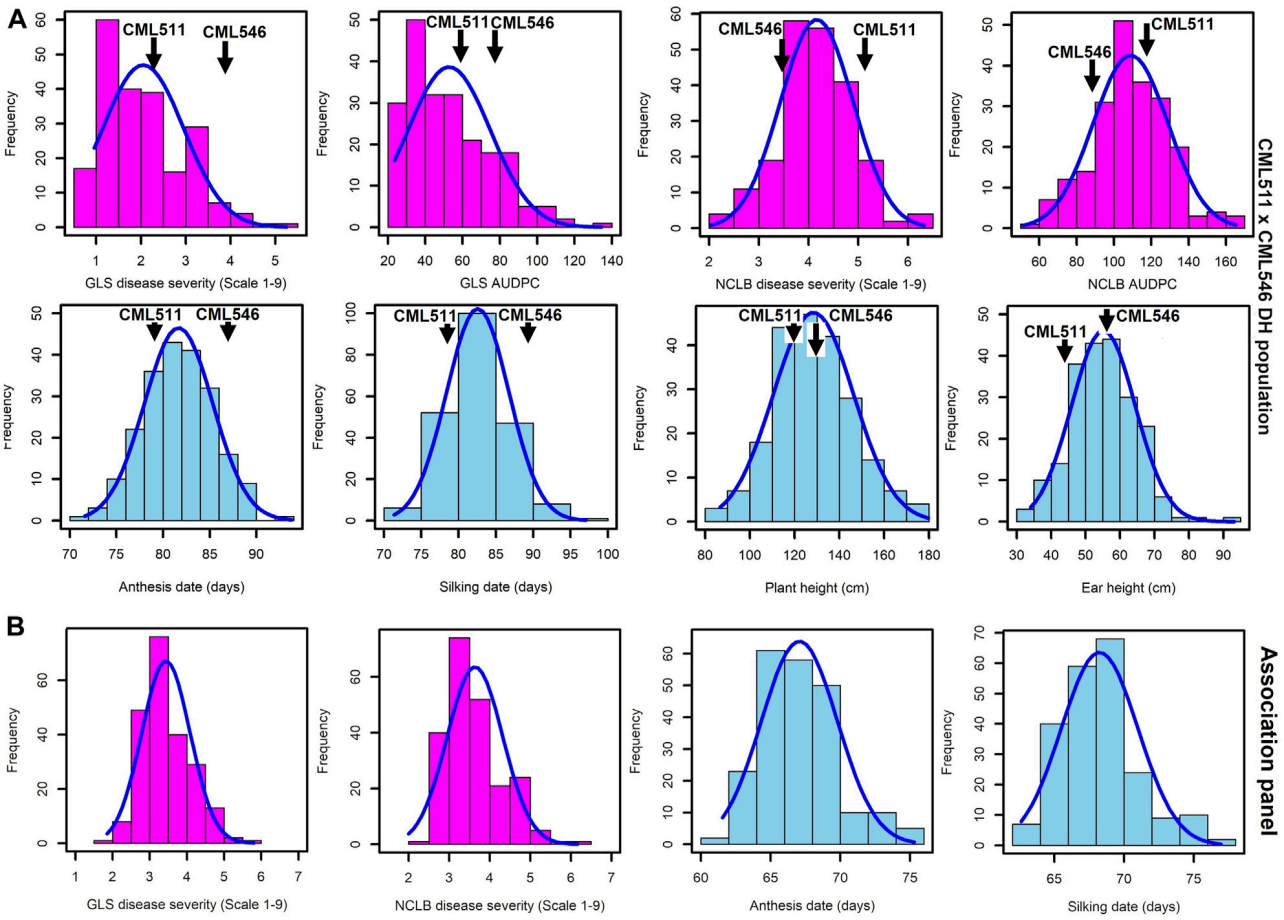
Frequency distributions for GLS, NCLB disease, and other agronomic traits, namely, anthesis date, silking date, plant height, and ear height, evaluated across the three locations in western Kenya. **(A)**. Biparental CML511×CML546 DH population of 230 lines. **(B)**. Association panel of 239 sub-tropical and tropical maize lines across the three locations. DS scores were for the last rating time point.

As expected for western Kenya, there was high natural disease pressure for both NCLB and GLS for all field trials of the biparental CML511×CML546 DH population (Figure 1A), as well as the association panel (Figure 1B). DS scores for both NCLB and GLS were highest at the final disease rating time point in all field trials, which corresponded in most cases to the last disease rating time point (Supplementary Table S1). A significant difference in resistance to NCLB was reported for the two parents CML511 and CML546 (*p-value* = 0.011831, *α* < 0.05). Our data show that CML511 is moderately susceptible and CML546 is more resistant to NCLB. On the other hand, the two parents differed slightly but not significantly in resistance to GLS (*p-value* = 0.200588, *α* < 0.05). For GLS, CML511 had an average score of 2.47 at the final rating, while CML546 had an average score of 4.0 at the final average DS score (Supplementary Figure S1). These evaluations show that CML511 is resistant and CML546 is moderately susceptible to GLS. A large portion of the biparental population was extensively blighted by NCLB. Transgressive segregation was observed in the population for GLS, NCLB, and AD, as some of the genotypes were more resistant or susceptible compared to the parental lines in the biparental population (Figure 1).

The frequency distribution of GLS DS scores was fairly skewed toward resistance in the biparental population (Figure 1A). The frequency distribution of the DS scores for NCLB in the biparental population followed an approximately normal distribution, as shown in Figure 1A. The wide segregation of DS and AUDPC scores for NCLB provided more evidence for the quantitative control of resistance. The DH population also exhibited continuous distribution for the days to anthesis, days to silking, plant height, and ear height (Figure 1A). The GLS and NCLB DS scores for the maize association panel ranged from 1.5 to 6 (Figure 1B), which were similar to the biparental DH population scores, although the association panel trended toward higher GLS and lower NCLB scores. On the other hand, the use of the nine-point rating scale revealed extensive phenotypic variation in resistance to GLS and NCLB across the association panel, with the panel harboring more resistant lines (Figure 1B). The association panel was also characterized by shorter days to anthesis compared to the biparental population (Figure 1B).

### 3.2 Correlations between environments and variables

In the DH population, the correlation between environments for GLS DS was positive and highly significant (*p* < 0.001) (Supplementary Table S2). A moderately high correlation was observed between environments for NCLB DS scores (Supplementary Table S2). The correlation across environments for AD and SD was also highly significant at *p* < 0.001 (Supplementary Table S2).

The analyses of variance revealed significant genotypic and genotype × environment interaction variances for GLS, NCLB DS, and AUDPC values as well as other agronomic traits (Table 1). Heritability estimates on an entry mean basis were 0.81, 0.81, 0.79, and 0.80 (Table 1) for GLS DS, AUDPC for GLS, NCLB DS, and AUDPC for NCLB, respectively in the DH population. However, the heritability estimates for DS in the association mapping panel were lower (0.35 for GLS and 0.64 for NCLB).

**TABLE 1:** Estimates of means, components of genotypic (σ^2^_G_), genotype × environment interaction (σ^2^_GxE_), error variances (σ^2^_e_), and heritability (h^2^) for the biparental CML511×CML546 DH population and the association panel evaluated across three environments each for GLS, NCLB, and other agronomic traits.

	AD	SD	PH	EH	GLS	GLS	NCLB	NCLB	GLS^a^	NCLB^a^
					DS	AUDPC	DS	AUDPC		
Mean	81.74	82.69	127.89	55.02	2.36	52.27	4.58	108.46	3.11	3.62
σ^2^_G_	11.07**	14.37**	282.87**	67.79**	0.94**	382.44**	0.42**	311.71**	0.03*	0.07**
σ^2^_GxE_	2.37**	3.63**	16.66**	5.94**	0.49**	213.07**	0.19*	156.61**	0.09**	0.04**
σ^2^_e_	11.59	12.6	219.57	101.49	0.52	175.63	0.45	216.91	0.16	0.17
h^2^	0.84	0.85	0.90	0.84	0.81	0.81	0.79	0.80	0.35	0.64
LSD_5%_	2.69	3.04	11.21	7.07	0.84	17.31	0.6	15.95	0.33	0.52
CV	4.17	4.29	11.59	18.31	30.56	25.36	14.73	13.58	34.09	18.06

AD, days to anthesis; SD, days to silking; PH, plant height; EH, ear height; DS, disease severity on a scale of 1–9; GLS, gray leaf spot; AUDPC, area under the disease progress curve; NCLB; northern corn leaf blight; CV, coefficient of variation; LSD, least significant difference; *h* ^*2*^, broad-sense heritability; *, **significant at *p*=0.05 and 0.01 levels, respectively.

^a^ Disease severity scores of the maize association panel.

**FIGURE 2:**
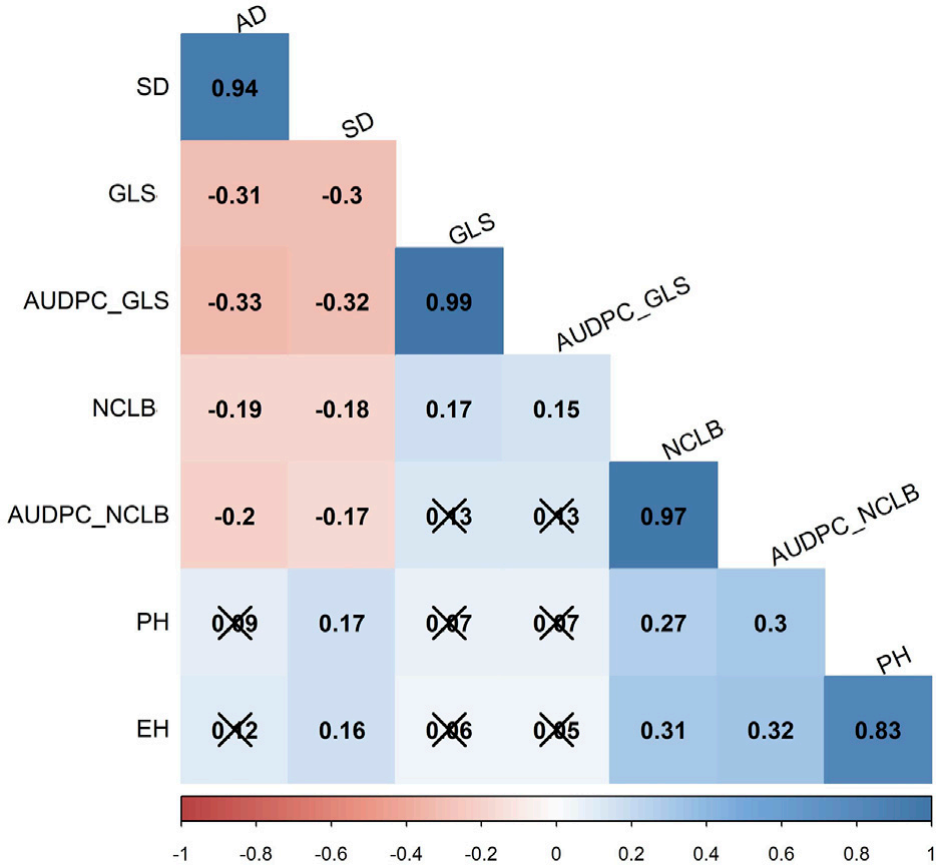
Pairwise Pearson correlation analysis for eight traits evaluated in three field trials in the biparental CML511×CML546 DH population. AD, anthesis date; SD, silking date; GLS, gray leaf spot, NCLB, northern corn leaf blight; AUDPC, area under disease progress curve; PH, plant height; and EH, ear height. The x marks indicate values that are not significant at *p* < 0.05.

**FIGURE 3:**
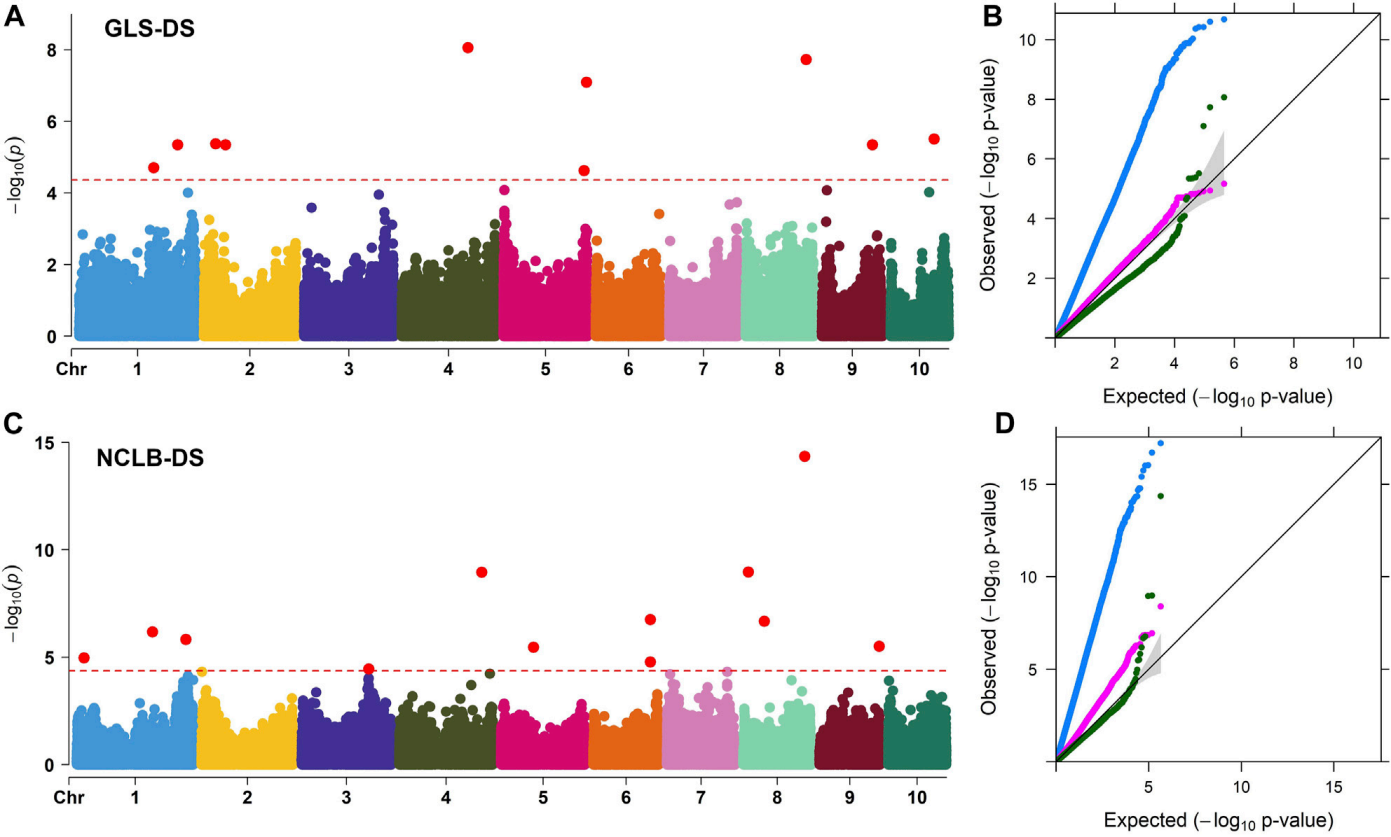
**(A, C)** Manhattan plots for the GWAS of GLS and NCLB disease severity in the maize association mapping panel. The dashed horizontal line of the Manhattan plot depicts the significance threshold value of *p* < 8 × 10^−5^. The x-axis indicates the SNP location along the 10 chromosomes, with chromosomes separated by different colors. Q–Q plots **(B, D)** of the estimated -log_10_(*p*) from association panel for GLS-DS and NCLB_DS traits. The black line bisecting the plot in Q–Q plots represents the expected *p*-values with no associations present. The blue line represents the observed *p*-values using the simplest model GLM(PCA + G) where the association between a phenotype and markers is directly detected. The pink line represents the observed *p*-values using the MLM (PCA + K + G) model. The green line represents the observed *p*-values using the FarmCPU model. G, genotype (fixed); PCA, three principal components (fixed); and K, kinship matrix (random).

Interestingly, the correlation analyses in the DH population showed low positive significant correlation between GLS (and AUDPC_GLS) and NCLB (Figure 2), indicating that there are different genomic loci that explain the variance for each disease. The agronomic traits for reproductive traits, namely, flowering time (AD) and days to silking (SD), were significantly negatively correlated with DS and AUDPC for both GLS and NCLB diseases (Figure 3). This indicated that maize lines with earlier maturity had higher DS. As expected, ear height (EH) was highly correlated with plant height (PH). GLS DS and AUDPC values were weakly correlated with PH and EH, whereas NCLB DS and AUDPC values were positively and significantly correlated with PH and EH (Figure 3). There were weak positive and significant correlations between SD and PH/EH (Figure 3).

### 3.3 Construction of the genetic linkage map and QTL analyses

The linkage map for the CML511×CML546 DH population was constructed with a total of 1,264 SNP markers. The genetic linkage map spanned a total map length of 3,344.9 cM with 2.65 cM as the average distance between two adjacent markers. The linkage map, as shown in Supplementary Figure S2, covered most of the maize genome.

Several QTLs associated with resistance to GLS and NCLB with small additive effects were detected through inclusive composite interval mapping. QTL analyses revealed 10 QTLs distributed on chromosomes 1, 2, 3, 5, 7, 9, and 10 for GLS DS which individually explained 2.6%–15.2% of phenotypic variance and together explained 64.19% of total phenotypic variance (Table 2). All 10 QTLs detected for GLS DS were also consistently detected for GLS AUDPC values (Supplementary Table S3). For NCLB DS, 13 QTLs distributed in all chromosomes except for chromosomes 9 and 10, individually explained 2.2%–8.7% which together contributed 64.94% of total phenotypic variation. AUDPC values for NCLB revealed nine QTLs together explained 45% of total phenotypic variance (Supplementary Table S3). Three QTLs on chromosomes 3, 6, and 7 were consistent across NCLB DS and AUDPC values. Among the agronomic traits, for AD, nine QTLs detected together explained 63% of the total phenotypic variance, and one QTL on chromosome 8 (*qAD8_137*) was a major effect QTL which explained 15.84% of phenotypic variance ((Supplementary Table S4). For SD, six QTLs were detected which together explained 60% of the total phenotypic variance. There were three QTLs on chromosomes 1, 4, and 8 that were consistent for both AD and SD. For PH, four QTLs were identified which together explained 52% of total phenotypic variance. One major effect QTL (*qPH8_129)* detected on chromosome 8 explained 22% of phenotypic variance. For EH, six minor effect QTLs and one major effect (*qEH8_128*) QTL were detected which together explained 56% of total phenotypic variance. QTL mapping predominantly revealed that the additive gene effect defined the gene action for resistance to GLS and NCLB.

**TABLE 2:** QTL detected by integrated composite interval mapping analysis for resistance to GLS and NCLB in the DH population evaluated in multiple locations.

Trait name	QTL name^a^	Chr	Position (cM)	Left marker^b^	Right marker^b^	LOD	PVE (%)	TPVE (%)	Add	Fav parent
GLS DS	*qGLS1_54*	1	163	S1_283894617	S1_53456776	8.95	5.33	64.19	0.22	CML546
	*qGLS1_186*	1	372	S1_190286762	S1_185978658	21.86	15.17		−0.28	CML511
	*qGLS1_185*	1	383	S1_185978658	S1_143231392	11.34	9.01		0.21	CML546
	*qGLS2_30*	2	208	S2_30710232	S2_32668550	3.79	2.24		0.11	CML546
	*qGLS3_151*	3	92	S3_157562360	S3_150546157	5.31	3.37		0.13	CML546
	*qGLS5_07*	5	189	S5_7548544	S5_1579511	3.62	2.25		−0.11	CML511
	*qGLS5_16*	5	284	S5_15869219	S5_23093956	5.76	3.56		−0.14	CML511
	*qGLS7_158*	7	105	S7_158889984	S7_158892468	4.49	2.58		−0.11	CML511
	*qGLS9_129*	9	154	S9_135788881	S9_129671108	4.27	3.53		−0.13	CML511
	*qGLS10_50*	10	217	S10_43765534	S10_54916081	3.19	6.21		0.22	CML546
NCLB DS	*qNCLB1_230*	1	70	S1_229375633	S1_232878545	6.20	4.27	64.94	0.12	CML546
	*qNCLB1_303*	1	429	S1_303106691	S1_304299395	3.13	2.08		0.08	CML546
	*qNCLB2_220*	2	164	S2_223388206	S2_32056786	5.40	3.82		0.16	CML546
	*qNCLB3_02*	3	163	S3_2734515	S3_1173815	4.95	3.57		0.11	CML546
	*qNCLB3_50*	3	185	S3_65853211	S3_12761976	9.74	8.76		0.18	CML546
	*qNCLB4_200*	4	272	S4_200040593	S4_201402668	3.50	4.58		−0.12	CML511
	*qNCLB5_83*	5	80	S5_82971208	S5_160085856	3.60	4.56		0.13	CML546
	*qNCLB5_195*	5	113	S5_194106967	S5_198705622	3.60	2.76		0.10	CML546
	*qNCLB6_137*	6	204	S6_136207036	S6_137005821	3.21	2.17		−0.09	CML511
	*qNCLB6_153*	6	335	S6_151834390	S6_153165363	3.95	3.58		−0.20	CML511
	*qNCLB7_120*	7	96	S7_121214712	S7_47406965	7.51	5.58		−0.14	CML511
	*qNCLB7_174*	7	257	S7_170932028	S7_174093748	3.43	2.44		−0.10	CML511
	*qNCLB8_171*	8	276	S8_170418369	S8_171776990	7.85	5.77		0.14	CML546

GLS DS, gray leaf spot disease severity; NCLB DS; northern corn leaf blight disease severity; LOD, logarithm of odds; add, additive effect; PVE, phenotypic variance explained; fav parent, parental genotype from where a favorable allele is contributing.

^a^ QTL, name composed by the trait code followed by the chromosome number in which the QTL was mapped and a physical location of the QTL. QTL names are italicized.

^b^ The exact physical position of the marker can be inferred from the marker’s name, for example, S1_82702920: chromosome 1; 82,702,920 bp (Ref Gen_v3 of B73).

### 3.4 GWAS analysis

After a quality check of GBS SNP markers, 230,743 SNPs were retained for the final association analyses (Supplementary Figure S3). The kinship matrix for these 239 lines was projected in the form of a heatmap which shows the magnitude of the relationship between the individuals (Supplementary Figure S4). This clearly showed that there is no strong population structure in the association panel used here. PCAs revealed five subpopulations within the panel (Supplementary Figure S5).

Association analyses for GLS DS and NCLB DS data were performed with GLM, MLM, and FarmCPU models (Figure 3). For both GLS and NCLB DS traits, for the GLM model, the observed *p*-values showed higher deviation from the expected *p*-values which may cause high false positives. On the other hand, for the MLM model, though the observed *p*-values were closer to the expected *p*-values, overfitting of the model is observed. For the FarmCPU model, the observed *p*-values were close to the expected *p* values and were more effective in controlling the false positives (Figure 3). The FarmCPU model is known to use both fixed and random effects models iteratively which helps in avoiding overfitting of the model by stepwise regression (Liu et al., 2016). Therefore, in this study, we used the FarmCPU model in the association mapping.

Association analyses revealed 11 and 18 SNPs significantly associated with GLS DS and NCLB DS, respectively (Figure 3; Table 3). For GLS-DS, the identified SNPs were distributed across all chromosomes except for chromosomes 3, 6, and 7 (Figure 3). The Manhattan plot revealed that for GLS DS, the highest peak was reported on chromosomes 4 and 8 (Figure 3A), while for NCLB DS, the highest peak was reported on chromosome 8 (Figure 3C). Furthermore, we determined whether any of these significant GLS or NCLB disease-associated SNPs identified in the association analysis is co-located with the QTL for GLS or NCLB in the biparental DH population. Two SNPs on chromosome 1 (*S1_192041854* and *S1_253381765*) were co-located with *qGLS1_54* detected for both GLS DS and AUDPC values in the DH population (Table 2; Table 3). Another SNP on chromosome 9 (*S9_130213878*) was found to be collocated within the *qGLS9_129* and *qG_AUDPC9_129* QTL region (Table 2; Table 3). Among the 16 SNPs identified for NCLB, marker *S5_83980678* is found to be within the region of NCLB DS QTL *qNCLB5_83* (Table 2; Table 3).

**TABLE 3:** Chromosomal position and SNPs significantly associated with GLS disease severity (GLS_DS) and northern corn leaf blight disease severity (NCLB-DS) detected by SNP-based GWAS in the association mapping panel.

SNP^a^	Chr	MLM *p*-value	MAF^b^	H&B^c^ *p*-value	Effect	Candidate gene	Gene annotation
**GLS-DS**							
S4_170027069	4	8.68E-09	0.35	0.00	0.16	*Zm00001eb189650*	K13120—protein FAM32A (FAM32A)
S8_155438805	8	1.86E-08	0.47	0.00	−0.20	*Zm00001eb360540*	Cation efflux protein
S5_214099678	5	7.99E-08	0.29	0.01	0.18	*Zm00001eb254100*	Zinc finger FYVE domain-containing protein
S10_112359288	10	3.09E-06	0.13	0.13	−0.20	*Zm00001eb421180*	Copper transport protein atox1-related (abiotic stress)
S2_29666484	2	4.25E-06	0.38	0.13	−0.13	*Zm00001eb077270*	Wall-associated receptor kinase galacturonan-binding domain (defense)
S1_253381765	1	4.51E-06	0.40	0.13	−0.13	*Zm00001eb049550*	Sel-1-like protein
S9_130213878	9	4.53E-06	0.10	0.13	−0.24	*Zm00001eb393380*	No associated annotations
S2_55483916	2	4.53E-06	0.26	0.13	0.13	*ZM00001EB083120*	No associated annotations
S1_192041854	1	1.98E-05	0.26	0.51	0.15	*Zm00001eb034870*	DNA damage–repair/toleration protein (plant defense)
S5_208091867	5	2.37E-05	0.21	0.55	0.12	*Zm00001eb251710*	Brevis radix domain/regulator of chromosome condensation (plant defense)
S5_2923669	5	8.36E-05	0.24	1.00	0.13	*Zm00001eb211960*	NAD-dependent epimerase/dehydratase//cinnamoyl-COA reductase-like
**NCLB-DS**							
S8_157881780	8	4.41E-15	0.15	0.00	−0.57	*GRMZM2G059590*	Uncharacterized protein LOC103636219
S8_12990030	8	1.07E-09	0.16	0.00	−0.27	*Zm00001d008560*	No associated annotations
S4_212315234	4	1.11E-09	0.16	0.00	0.25	*Zm00001eb201110*	ATP-binding cassette transporter//ABC transporter (plant defense)
S6_147054037	6	1.75E-07	0.08	0.01	0.25	*Zm00001eb285130*	TPR repeat-containing THIOREDOXIN TDX
S8_54094984	8	2.11E-07	0.49	0.01	−0.17	*Zm00001eb341620*	Thioesterase superfamily member-related
S1_194762510	1	6.65E-07	0.22	0.03	−0.26	*Zm00001eb035640*	AUX/IAA protein//B3 DNA-binding domain//auxin response factor//DNA-binding pseudobarrel domain (plant defense)
S1_280826386	1	1.48E-06	0.28	0.05	0.17	*Zm00001d034003*	Seed maturation family protein
S9_153843575	9	3.08E-06	0.45	0.09	0.16	*Zm00001eb400490*	Pre-mRNA-processing factor
S5_83980678	5	3.39E-06	0.14	0.09	0.22	*Zm00001eb232660*	Helicase superfamily/ATP-binding domain (plant defense)
S1_18630129	1	1.05E-05	0.45	0.24	−0.13	*Zm00001eb006570*	WD and tetratricopeptide repeats protein 1 (WDTC1)
S6_146813774	6	1.64E-05	0.06	0.34	−0.21	*Zm00001eb285080*	Protein kinase domain (plant defense)
S3_173387708	3	3.49E-05	0.09	0.67	−0.21	*Zm00001eb144960*	Lipoxygenase (plant defense)
S2_923555	2	4.77E-05	0.32	0.79	−0.16	*Zm00001d001787*	Cleavage and polyadenylation specificity factor subunit 5 (plant defense)
S7_155701108	7	4.77E-05	0.14	0.79	0.21	*Zm00001d021552*	Protein of unknown function
S4_233626821	4	5.87E-05	0.47	0.88	−0.15	*Zm00001eb204230*	Voltage- and ligand-gated potassium channel
S7_8047716	7	6.13E-05	0.24	0.88	−0.12	*Zm00001d018877*	Plastocyanin-like domain (Cu_bind_like)

^a^ The exact physical position of the SNP can be inferred from the marker’s name, for example, S5_51353429: chromosome 5; 51353429 bp (Ref Gen_v2 of B73).

^b^ Minor allele frequency. Candidate gene names are italicized.

^c^ False discovery rate calculated by using the Benjamini and Hochberg correction method.

To elucidate the molecular and physiological mechanisms controlling GLS and NCLB DS, candidate genes were identified. On all chromosomes, a total of 24 candidate genes were discovered (Table 3). Four candidate genes closely associated with the SNPs for GLS resistance were identified, namely, *Zm00001eb077270*, *Zm00001eb034870*, *Zm00001d017831*, and *Zm00001eb211960* (Table 3). There were six candidate genes with defense response annotations that were associated with SNPs for NCLB resistance (*Zm00001eb201110*, *Zm00001eb035640*, *Zm00001eb232660*, *Zm00001eb285080*, *Zm00001eb144960*, and *Zm00001d001787*) (Table 3).

**FIGURE 4:**
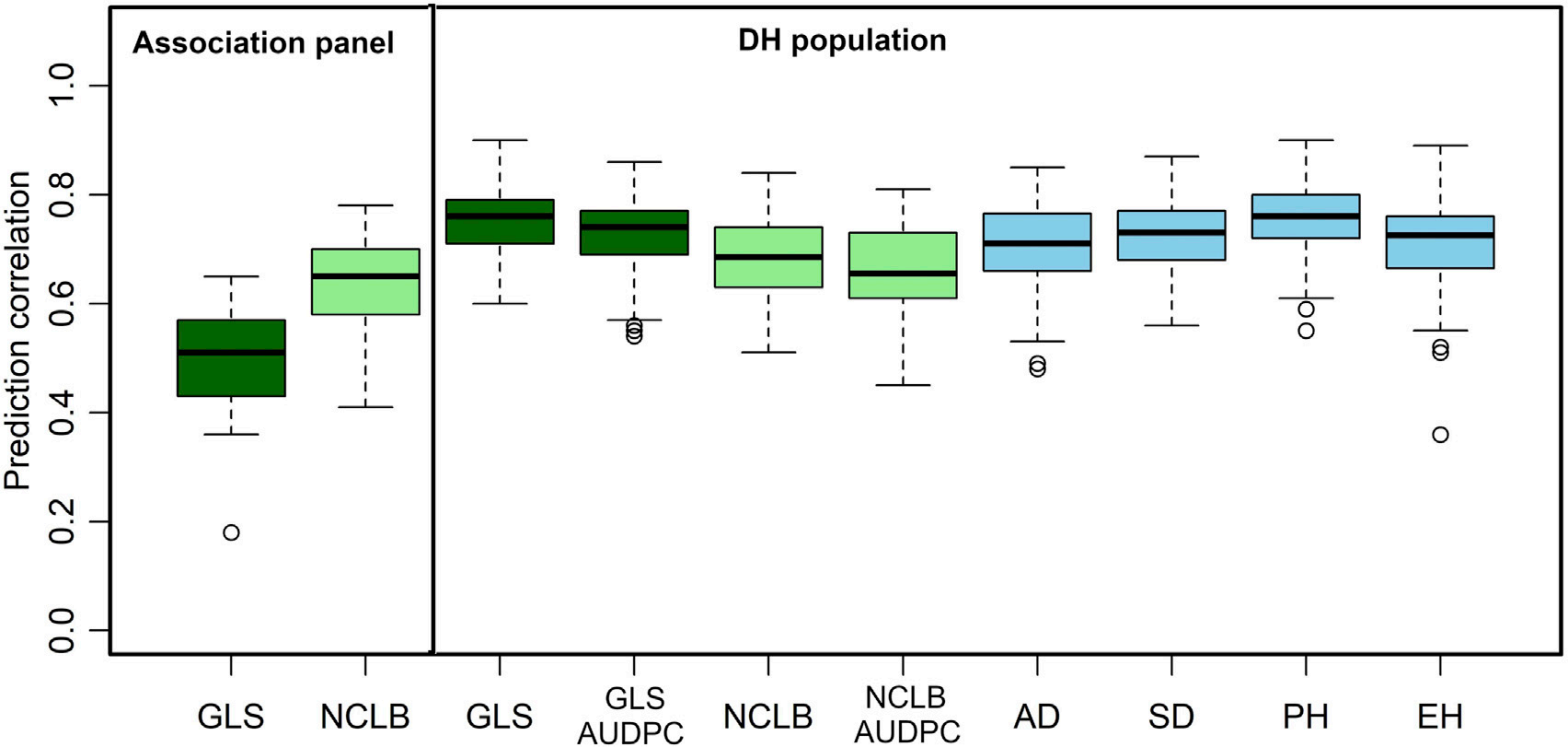
Box-whisker plots for the accuracy of genomic predictions assessed by five-fold cross-validation within association and DH population. AD, days to anthesis; SD, days to silking; PH, plant height; EH, ear height; GLS, gray leaf spot; AUDPC, area under the disease progress curve; NCLB, northern corn leaf blight.

Genomic prediction captures all variations from small to large effects, which helps in improving complex traits. Prediction correlations obtained from cross-validations are commonly used to know the effectiveness of genomic predictions for different traits. In this study, we applied genomic predictions within the DH population and association panel for disease traits and also for agronomic traits (Figure 4).

As expected, the mean prediction correlations for the DS traits were higher in the DH population (GLS DS = 0.75, NCLB DS = 0.68) than those in the association panel (GLS DS = 0.63, NCLB = 0.49) (Figure 4). This was because of the highly relatedness among the DH lines compared to the lines in the association panel. In addition, relatively high-average prediction correlations were obtained for the other traits when validated in the DH population, namely, 0.73, 0.66, 0.71, 0.72, 0.75, and 0.71 for GLS-AUDPC, NCLB-AUDPC, AD, SD, PH, and EH, respectively.

## 4 Discussion

GLS and NCLB are economically important foliar diseases of maize. Understanding their genetic basis of resistance is valuable to designing an effective breeding strategy (Beyene and Prasanna, 2020). The DH population used in this study was artificially inoculated with GLS; however, the locations evaluated were also a hotspot for NCLB, so we observed both GLS and NCLB disease symptoms in the same population. For early scoring, symptoms for both diseases were clearly distinguishable, which helped to score the data with more accuracy. Scoring at a later stage of disease development was tricky due to bigger blight merging with leaf spots, so for the analyses, we used the third DS score for both GLS and NCLB. The association mapping panel was also evaluated in natural hot spots for GLS and NCLB, and scoring was performed only once at a grain-filling stage when clearly distinguished GLS and NCLB symptoms were observed. Therefore, the collected DS data represent the real response of these lines to the respective diseases. Nevertheless, both diseases appearing at the same growth stage and in the same experiment can lead to some confounding effects. Most of the lines in both the DH population and the association panel fall into the categories of resistant and moderately resistant, with a few in moderately susceptible but none in the completely susceptible category (Figure 1)*.* Overall, the phenotypic data in this study showed a normal distribution for NCLB and GLS DS scores in both the biparental CML511×CML546 DH population and the association panel which supports the quantitative nature of resistance in these diseases (Nyanapah et al., 2020). The parental line CML511 exhibited a moderate level of resistance to GLS congruent with the observations of Kibe et al. (2020a). We also observed significant genotypic and genotype × environment interaction variance and moderate-to-high heritabilities in both the DH population and the association panel, indicating good prospects for introgressing GLS and NCLB resistance in breeding programs. Observed heritability estimates for GLS, NCLB, and other agronomic traits in the DH population are consistent with earlier studies (Wisser et al., 2011; Benson et al., 2015).

### 4.1 Trait correlations

There were moderate correlations in GLS and NCLB DS scores for the biparental DH population between the field trials, indicating that trait expression was relatively consistent between the evaluated locations (Supplementary Table S2). On the other hand, a significantly negative correlation was observed between DS data of GLS and NCLB, with the flowering time traits AD and SD in this study (Figure 2). This implies that lower values for AUDPC (implying higher levels of disease resistance) corresponded with longer AD or SD. Such negative correlations have also been reported in other studies (Asea et al., 2009; Wisser et al., 2011; Kolkman et al., 2020). On the contrary, some studies reported a positive correlation between GLS resistance and flowering time (Balint-Kurti et al., 2008; Zwonitzer et al., 2010; Benson et al., 2015; Mammadov et al., 2015; Liu et al., 2016). This suggests the cautious use of flowering time in the selection of lines for resistance to GLS and NCLB.

In Kenya and Uganda, the main maize-growing area is frequently affected by GLS and NCLB pathogens (Borchardt et al., 1998; Nsibo et al., 2021). When both pathogens affect the maize at the same time, more pronounced necrotic symptoms are the major concern which are probably due to the synergistic interactions of both pathogens. However, a weak correlation was observed between GLS DS and NCLB DS. One of the QTLs identified for GLS (*qGLS2_30*) was in proximity with the QTL identified for NCLB (*qNCLB2_220*). In the comparison of SNPs associated with GLS and NCLB DS in association mapping, it was observed that two SNPs (*S1_194762510* and *S1280826386)* associated with NCLB DS were collocated within GLS QTL (*qGLS1_54*) (Tables 2, 3). This suggests that there are some common regions contributing to resistance for both diseases. On the other hand, the observed weak correlation between the DS of the two diseases could be attributed to the different infection strategies of the associated pathogens. *Cercospora zeina* is an apoplastic necrotroph and a hemibiotroph, while *E. turcicum* is apoplastic but then enters the vascular system of the leaf (Kotze et al., 2019). They also exploit different pathogenicity factors in causing disease symptoms (Swart et al., 2017; Human et al., 2020).

### 4.2 QTLs associated with GLS resistance

Most of the QTLs detected for GLS DS were also detected for GLS AUDPC (Table 2). This was well supported by the observed strong correlation (r = 0.99) between GLS DS and GLS AUDPC (Figure 2). A major QTL for GLS resistance (DS and AUDPC), *qGLS1_186*, which explained 15.16% of the phenotypic variance, overlapped with *qGLS1_185* which also explained 9.01% of the phenotypic variance. Intriguingly, this major QTL has favorable alleles from the donor parent CML511. Kibe et al. (2020a) using the CML550×CML511 DH population also detected major effect QTL *qGLS1-155* which was located within the physical position of 154–157 Mbp which overlapped with the QTL detected in the present study (*qGLS1_185*) spanning between 143 and 185 Mbp. Sun et al. (2021) also fine-mapped a major effect QTL at the 187–189 Mb region, and the reported flanking markers would be useful to validate in tropical germplasm.

The chromosome bin 1.06 was described as a QTL hotspot for GLS resistance as many studies reported earlier (Lehmensiek et al., 2001; Shi et al., 2007; Balint-Kurti et al., 2008; Berger et al., 2014; He et al., 2018; Lopez-Zuniga et al., 2019; Sun et al., 2021). The chromosome bin 1.06 also harbors resistance genes to several other diseases like common rust, southern leaf blight (SLB), ear rot, and NCLB (Freymark et al., 1993; Wisser et al., 2006; Chung et al., 2010b; Zwonitzer et al., 2010; Poland et al., 2011; Jamann et al., 2015). Chung et al. (2010b) demonstrated that the NCLB resistance QTL at bin 1.06 was important to protect the host against fungal penetration of *E. turcicum* using an introgression line population. The chromosomal region has also been associated with effects on diverse traits such as grain yield and its components, anthesis silking interval, and root and shoot traits under both water stress and optimal water environments (Ribaut et al., 1996; Tuberosa et al., 2002; Landi et al., 2010). The concentrated mapping of QTL for several traits, including multiple disease resistance in this chromosomal region, provides breeders and geneticists an opportunity to dissect them further and find tightly linked flanking markers so that this region can be utilized to develop cultivars with multiple disease resistance.

The *qGLS2_30* QTL identified in this study in the chromosomal bin 2.04 overlapped with QTL reported in earlier studies using different mapping populations (Balint-Kurti et al., 2008; Lennon et al., 2017). The QTL *qGLS3_151* is placed between 150 and 157 Mbp in the chromosomal bin 3.05, which has previously been identified as conditioning resistance to SLB and GLS (Zwonitzer et al., 2010; Kump et al., 2011). The QTL *qGLS7_158* positioned between 157 and 159 Mbp in the chromosomal bin 7.04 was also previously reported by Berger et al. (2014) for GLS resistance. Another QTL *qGLS5_16* in the chromosomal bin 5.03 is also known to have several reported markers for GLS resistance in a number of association mapping studies (Bubeck et al., 1993; Clements et al., 2000; Lehmensiek et al., 2001; Shi et al., 2007; Zhang et al., 2012; Benson et al., 2015). Overall, many of the QTLs detected in the present study overlapped between the biparental CML511 × CML546 DH population and the association panel as well as earlier studies (Supplementary Table S5). The major effect QTL for both GLS on chromosome 1 is of immediate interest to be used in resistance breeding.

Among the nine candidate genes identified for GLS resistance through association mapping, one on chromosome 2 (*Zm00001eb077270*) encodes a putative receptor-like protein kinase which are transmembrane signaling proteins that are able to sense changes in the extracellular environment such as pathogen invasion (Decreux et al., 2006; Qi et al., 2023). Another candidate gene on chromosome 1 (*Zm00001eb034870*) encodes DNA damage–repair/toleration protein that harbors a leucine-rich repeat domain which serve as the first line of defense in response to pathogen-associated molecular patterns (Ng and Xavier, 2011). The candidate gene *Zm00001eb144960* on chromosome 3 encodes lipoxygenases which are known to be associated with pest resistance, response to wounding, and plant defense mechanisms where it was reported to be involved in the early response to pathogen attack (Peng et al., 1994). Overall, the detected candidate genes in the association study have annotations inferring direct or indirect involvement in plant defense.

### 4.3 QTL and SNPs associated with resistance to NCLB

This study identified 13 QTLs for NCLB DS and nine QTLs for NCLB AUDPC. Three of these QTLs were common for both the DS and AUDPC NCLB traits. The first example of this was QTLs *qNCLB3_50* and *qN_AUDPC3_50* that co-localized in the same position in bin 3.04. This significant QTL for NCLB resistance has favorable alleles from the parent CML546 (the more resistant parent). Previous research reports also identified bin 3.04 as a QTL hotspot conditioning resistance to multiple diseases including NCLB, SLB, and GLS (Lehmensiek et al., 2001; Wisser et al., 2006; Shi et al., 2007; Zwonitzer et al., 2010; Kump et al., 2011; Liu et al., 2016; Lennon et al., 2017; Martins et al., 2019).

The QTL *qNCLB5_83* was positioned in the chromosomal bin 5.04. According to Miedaner et al. (2020), up to eight QTLs have been localized in this bin showing the importance of this region for NCLB resistance. Interestingly, the SNP identified through association mapping (*S5_83980678*) is positioned within this QTL region (Table 2, 3). It is associated with a candidate gene (*Zm00001eb232660*) that encodes a DNA helicase/ATP-binding domain. This type of domain has a catalytic function in unwinding of the double-stranded DNA that is instrumental in the repair of damaged DNA and DNA replication (Koonin, 1993).

Similarly, *qNCLB6_153* for NCLB DS overlapped with *qN_AUDPC6_153* for the AUDPC on chromosome 6 (bin 6.05). Up to four QTLs were reported from different studies in the same region (Miedaner et al., 2020). Another pair of QTL, *qNCLB8_171* and *qN_AUDPC8_171*, corresponded with a previously reported NCLB resistance QTL by Galiano-Carneiro et al. (2021). Interestingly, none of the NCLB DS QTLs detected in this study were found in the same position with chromosomal bins associated with the qualitative *Ht* genes (Galiano-Carneiro and Miedaner, 2017).

An association study on NCLB revealed a significant marker linked to a candidate gene *Zm00001eb201110* on chromosome 4 which encodes for an ATP-binding cassette (ABC) transporter. Plant proteins with this function are known to be associated with resistance to fungal and bacterial pathogens through the transmembrane transport of jasmonic acid or antimicrobial secondary metabolites (Zhang et al., 2020). Using GWAS, many studies showed the association of ABC transporter genes with NCLB resistance (Poland et al., 2011; Ding et al., 2015). Another candidate gene, *Zm00001eb285080*, on chromosome 6 encodes a protein kinase, a function known to be important in regulating the response of plants to pathogen attack (Lehti-Shiu and Shiu, 2012). There is strong evidence that protein kinases play a pivotal role in resistance to NCLB (Poland et al., 2011; Ding et al., 2015; Kolkman et al., 2020).

### 4.4 QTLs associated with agronomic data

The major QTL for flowering time *qAD8_137* was collocated with QTLs *qSD8_137* for SD, *qPH8_129* for PH, and *qEH8_128* for EH ((Supplementary Table S4). These QTLs also explained the major effect of phenotypic variance of 15.8%, 21.4%, 22.39%, and 22.98% for AD, SD, PH, and EH, respectively. Several studies also recognized chromosomal bin 8.05 as a hotspot for flowering time QTL and genes (Balint-Kurti et al., 2008; Buckler et al., 2009; Van Inghelandt et al., 2012). Interestingly, two qualitative resistance genes, *Ht2* and *Htn1*, were also detected on the same chromosomal bin 8.05 (Galiano-Carneiro and Miedaner, 2017; Hurni et al., 2015). The genetic mechanisms underlying flowering time in this study were largely characterized by additive gene action. These results agree with the findings of Buckler et al. (2009) who reported that variations in days to flowering are due to the joint effect of many minor QTLs with additive effect.

Intriguingly, some of the QTLs associated with flowering time overlapped with the NCLB and GLS resistance QTL. For instance, *qAD1_60* for flowering time shared the same flanking markers as *qGLS1_54* (Table 2; Supplementary Table S4), and the two SNPs (*S1_192041854, S1_253381765*) for GLS DS and two SNPs (*S1_194762510, S1_2800826386*) for NCLB DS detected through association mapping are also positioned in this region. Another QTL *qGLS9_129* also had the same flanking markers as *qAD9_130*, and one SNP from association mapping (*S9_130213878*) was also detected in the same region for GLS DS. The NCLB QTL *qNCLB1_230* overlapped with *qAD1_227* on the maize chromosomal bin 1.07 (Table 2; Supplementary Table S4). The QTLs *qN_AUDPC2_188* and *qPH2_176* overlapped on chromosomal bin 2.06, sharing the flanking markers. This was further supported by the positive correlation between PH and NCLB AUDPC (Figure 2). On the other hand, Galiano-Carneiro et al. (2021) reported a negative correlation between PH and NCLB DS. There were no common QTL regions identified for PH and EH that spanned the same chromosomal regions as GLS DS and AUDPC. This is supported by the observed negative correlation between the two traits (Figure 3).

### 4.5 Genomic prediction of disease and agronomic traits

Compared to the association panel, the high-prediction correlations in the DH population for GLS and NCLB could be attributed to higher similarity or relatedness of individuals between the training set and the prediction set (Figure 4; Lorenz and Smith, 2015). GLS DS and GLS AUDPC exhibited slightly higher prediction accuracies compared to NCLB DS and other agronomic traits. Agronomic traits, such as AD, SD, PH, and EH, are characterized by more complex genetic networks, under the control of numerous QTLs, and affected by the influence of the environment (Wallace et al., 2014). This presents a challenge in improving them through phenotypic selection (Du et al., 2021). Kibe et al. (2020a) reported low-to-moderate prediction correlations within populations and high values when different related populations were combined and used in prediction. Similarly, Galiano-Carneiro et al. (2021) reported prediction accuracies of moderate levels (0.55) for prediction within families. Technow et al. (2013) reported prediction accuracies of 0.58 and 0.55 when using a small population size of 75 individuals. It has been reported that there is no difference among hybrids advanced through the genomic selection or phenotypic selection in their response to NCLB and GLS, with the genomic selection being relatively cheaper than the phenotypic selection (Beyene et al., 2019; 2021). Overall, the use of genomic selection has potential to improve the resistance to GLS and NCLB in breeding populations and could lead to the development of multiple disease-resistant lines and hybrids.

## 5 Conclusion

GLS and NCLB are the major biotic stresses that hinder maize production in high-yielding maize-growing areas in East Africa, such as western Kenya. The use of genomic tools can provide useful information to fast track the development of disease-resistant varieties. In this study, we aimed to identify and validate genomic regions associated with GLS and NCLB resistance in biparental and association mapping populations evaluated in multiple locations in western Kenya. We identified 10 and 11 QTLs for GLS resistance and 18 and 16 QTLs for NCLB resistance in the DH population and association mapping population, respectively. We detected a major QTL for GLS resistance, *qGLS1_186*, which explained 15.2% phenotypic variance and *qNCLB3_50* for NCLB resistance, explaining 8.8% of the phenotypic variance. Several common QTL regions between linkage mapping and association mapping and between NCLB and GLS AUDPC traits were detected. A negative correlation between flowering time and severity of the two diseases was reported. Several QTLs identified in the present study were also co-localized with the QTL previously mapped for GLS and NCLB resistance. Our study highlights that the combined use of linkage mapping and genomic selection is an effective strategy for the improvement of resistance. Genomic prediction sheds light on new ways to improve breeding for disease resistance with optimum allocation of resources and lays the foundation for a new era of resistance breeding.

## Data Availability

The datasets presented in this study can be found in online repositories: data.cimmyt.org/dataset.xhtml?persistentId=hdl:11529/10548956 and zenodo.org/records/10046213.
