# Supplementary material for: Combination of linkage and association mapping with genomic prediction to infer QTL regions associated with gray leaf spot and northern corn leaf blight resistance in tropical maize
Source: Front Genet. 2023 Nov 7;14:1282673. doi: 10.3389/fgene.2023.1282673 (PMC10661943; doi:10.3389/fgene.2023.1282673)
Supplement: Supplementary file 1 [file DataSheet2.pdf]

Supplementary Table S1. Averages for the different response variables for the parents CML511 and CML546 of the bi-parental mapping population across the three trials (environments).

Genotype	CML511	CML546
Ke_DTA	94.10	100.65
Ke_DTS	94.64	102.83
Ke_PH	133.93	133.84
Ke_EH	67.11	60.15
Ke_GLS1	1.06	1.05
Ke_GLS2	1.30	1.05
Ke_GLS3	1.32	1.32
Ke_GLS4	1.90	1.62
Ke_GLS5	1.92	1.92
Ke_GLS6	2.53	2.81
Ke_AUDPC1	49.76	47.75
Ke_Turc1	3.25	2.98
Ke_Turc2	3.59	3.02
Ke_Turc3	4.32	3.28
Ke_Turc4	4.30	3.31
Ke_Turc5	4.60	3.64
Ke_Turc6	4.69	3.85
Ke_AUDPC2	121.77	93.41
M2_DTA	72.18	75.59
M2_DTS	72.09	76.63
M2_PH	119.71	134.55
M2_EH	54.24	52.09
M2_GLS1	2.01	2.52
M2_GLS2	2.39	3.11
M2_GLS3	2.17	3.74
M2_GLS4	3.66	4.71
M2_GLS5	3.08	5.99
M2_AUDPC1	73.33	111.37
M2_Turc1	1.95	2.51
M2_Turc2	3.87	3.50
M2_Turc3	3.83	3.56
M2_Turc4	4.40	3.83
M2_Turc5	5.01	4.14
M2_AUDPC2	103.86	98.62
M3_DTA	78.50	80.49

DTA	78.41	80.90
M3_DTS	78.88	81.83
M3_PH	114.94	112.48
M3_EH	45.71	34.57
M3_GLS1	1.33	1.88
M3_GLS2	1.89	2.25
M3_GLS3	1.97	2.53
M3_GLS4	2.01	2.90
M3_GLS5	2.31	3.48
M3_AUDPC1	54.45	73.89
M3_Turc1	2.17	1.88
M3_Turc2	3.45	2.33
M3_Turc3	3.64	3.54
M3_Turc4	4.70	3.50
M3_Turc5	5.11	3.94
M3_AUDPC2	110.28	86.51

Supplementary Table S2. Correlation coefficients between environments for Gray Leaf Spot, Northern Corn Leaf Blight and Flowering Time across multi-environments.

	Gray leaf spot		Northern corn leaf blight	
Environment	Kabianga	Maseno LR 2018	Kabianga	Maseno LR 2018
MasenoLR2018	0.58***		0.61***	
MasenoLR2019	0.57***	0.82***	0.46***	0.56***
	Days to anthesis		Days to silking	
MasenoLR2018	0.68***		0.7156***	
MasenoLR2019	0.62***	0.73***	0.65***	0.74***

*** implies that the correlation coefficient was significant at $p < 0.001$.

Supplementary Table S3: Quantitative trait loci (QTL) detected by integrated composite intervals mapping analysis for area under disease progress curve (AUDPC) for GLS and NCLB in DH population evaluated in multiple locations.

Trait Name	QTL name[1]	Chr	Position (cM)	Left Marker[2]	Right Marker[2]	LOD	PVE (%)	TPVE (%)	Add	Fav parent
GLS AUDPC	*Qg_AUDPC1_54*	1	163	S1_283894617	S1_53456776	8.73	5.32		6.43	CML546
	qG_AUDPC1_186	1	372	S1_190286762	S1_185978658	21.15	14.97		-7.92	CML511
	qG_AUDPC1_185	1	383	S1_185978658	S1_143231392	11.77	9.42		6.26	CML546
	qG_AUDPC2_30	2	208	S2_30710232	S2_32668550	4.18	2.54		3.26	CML546
	qG_AUDPC3_151	3	91	S3_157562360	S3_150546157	5.13	3.29	64.49	3.79	CML546
	qG_AUDPC5_07	5	188	S5_7548544	S5_1579511	4.04	2.40		-3.21	CML511
	qG_AUDPC5_16	5	284	S5_15869219	S5_23093956	6.20	3.98		-4.14	CML511
	qG_AUDPC7_158	7	105	S7_158889984	S7_158892468	5.20	3.12		-3.64	CML511
	qG_AUDPC9_129	9	155	S9_129671108	S9_38368264	3.88	2.35		-3.12	CML511
NCLB AUDPC	*qN_AUDPC1_240*	1	90	S1_239142914	S1_244605862	4.28	4.79		-3.75	CML511
	qN_AUDPC2_188	2	77	S2_186201459	S2_188131029	3.49	3.78		3.32	CML546
	qN_AUDPC2_38	2	213	S2_38106170	S2_37959899	3.54	3.94		3.38	CML546
	qN_AUDPC3_50	3	185	S3_65853211	S3_12761976	3.23	4.32		3.62	CML546
	qN_AUDPC3_12	3	193	S3_12761976	S3_9360961	5.43	6.49	45.79	-4.38	CML511
	qN_AUDPC5_70	5	73	S5_69767339	S5_73930457	4.35	7.02		-4.51	CML511
	qN_AUDPC6_153	6	335	S6_151834390	S6_153165363	4.89	6.59		-7.75	CML511
	qN_AUDPC7_120	7	97	S7_121214712	S7_47406965	4.04	4.25		-3.54	CML511
	qN_AUDPC8_171	8	275	S8_170418369	S8_171776990	4.79	5.69		4.25	CML546

GLS; gray leaf spot, AUDPC- area under the disease progress curve, NCLB; Northern corn leaf blight; LOD = logarithm of odds; Add = Additive effect; PVE = phenotypic variance explained; fav parent = parental genotype from where a favourable allele is contributing; [1] QTL name composed by the trait code followed by

the chromosome number in which the QTL was mapped and a physical location of the QTL; [2] The exact physical position of the marker can be inferred from marker's name, for example, S1_82702920: chromosome 1; 82,702,920 bp (Ref Gen_v3 of B73).

Supplementary Table S4: Quantitative trait loci (QTL) detected by integrated composite interval mapping analysis for agronomic traits namely, anthesis date (AD), silking date (SD), plant height (PH) and ear height (EH) in DH population evaluated in three locations.

Trait Name	QTL name	Chr	Position (cM)	Left Marker	Right Marker	LOD	PVE (%)	TPVE (%)	Add	Fav parent
AD	*qAD1_227*	1	52	S1_228064911	S1_217179487	3.51	3.55	63.73	-0.66	CML511
	qAD1_60	1	134	S1_283894617	S1_53456776	10.21	8.36		0.99	CML546
	qAD2_213	2	151	S2_213714960	S2_213287172	4.60	3.02		0.60	CML546
	qAD3_200	3	34	S3_205591571	S3_189204947	5.32	3.40		0.65	CML546
	qAD4_18	4	155	S4_19769820	S4_17279467	3.61	2.46		-0.58	CML511
	qAD4_220	4	289	S4_214489216	S4_242283166	12.25	8.95		-1.03	CML511
	qAD6_150	6	70	S6_157597489	S6_34950594	4.96	3.28		0.62	CML546
	qAD8_137	8	250	S8_136861251	S8_138560361	20.45	15.84		1.37	CML546
	qAD9_130	9	151	S9_135788881	S9_129671108	3.87	3.53		0.64	CML546
SD	*qSD1_227*	1	65	S1_225916227	S1_229375633	9.41	7.82	60.19	-1.04	CML511
	qSD4_220	4	290	S4_214489216	S4_242283166	5.91	5.06		-0.84	CML511
	qSD6_110	6	110	S6_115434802	S6_106077660	3.55	3.08		0.65	CML546
	qSD6_90	6	324	S6_101722172	S6_88269305	3.06	4.32		1.46	CML546
	qSD7_48	7	97	S7_121214712	S7_47406965	3.61	2.84		0.63	CML546
	qSD8_137	8	249	S8_139048847	S8_136861251	22.63	21.44		1.72	CML546
PH	*qPH1_03*	1	182	S1_2881853	S1_5532836	3.68	4.17	52.75	-3.89	CML511
	qPH1_28	1	268	S1_28106472	S1_27326414	5.12	7.29		-5.13	CML511

	qPH2_176	2	68	S2_175507568	S2_186201459	6.46	6.30		-4.78	CML511
	qPH8_129	8	243	S8_128738672	S8_129741294	23.90	22.39		8.99	CML546
	qEH1_20	1	219	S1_19920008	S1_21989679	3.64	3.12		-1.42	CML511
	qEH1_53	1	280	S1_70621025	S1_52680413	3.24	2.73		1.36	CML546
	qEH2_165	2	62	S2_170427602	S2_164596994	3.82	3.36		1.47	CML546
EH	*qEH2_234*	2	111	S2_233445021	S2_242048658	3.67	3.43	56.12	-1.51	CML511
	qEH6_35	6	88	S6_34950594	S6_93095112	4.69	4.32		1.67	CML546
	qEH8_128	8	242	S8_128422019	S8_128738672	21.84	22.98		3.85	CML546
	qEH9_140	9	21	S9_139755085	S9_154594758	9.29	15.96		-4.70	CML511

AD - days to anthesis; SD-days to silking; PH -Plant height; EH-Ear height; LOD = logarithm of odds; Add = Additive effect; PVE = Phenotypic variance explained; fav parent = parental genotype from where a favourable allele is contributing; [1] QTL name composed by the trait code followed by the chromosome number in which the QTL was mapped and a physical position of the QTL; [2] The exact physical position of the marker can be inferred from marker's name, for example, S1_82702920: chromosome 1; 82,702,920 bp (Ref Gen_v3 of B73).

Supplementary Table S5: The common genomic regions identified across linkage mapping and association study for GLS and NCLB disease severity.

Trait Name	QTL name[1]	Chr	Physical interval (Mbp)	SNP[a]	Candidate gene
	qGLS1_54	1	53 - 283	S1_253381765	*Zm00001eb049550*
	qGLS1_186	1	185 - 190	S1_192041854	*Zm00001eb034870*
GLS DS	*qGLS2_30*	2	30 - 33	S2_29666484	*Zm00001eb077270*
	qGLS5_07	5	1.5 - 7.5	S5_2923669	*Zm00001eb211960*
	qGLS9_129	9	129 - 136	S9_130213878	*Zm00001eb393380*
NCLB DS	*qNCLB5_83*	5	82 - 160	S5_83980678	*Zm00001eb232660*

GLS - gray leaf spot; DS – disease severity; NCLB - Northern corn leaf blight; Mbp = Mega base pair; [a]The exact physical position of the marker can be inferred from marker's name, for example, S1_82702920: chromosome 1; 82,702,920 bp (Ref Gen_v2 of B73).
