# Supplementary material for: Combination of linkage and association mapping with genomic prediction to infer QTL regions associated with gray leaf spot and northern corn leaf blight resistance in tropical maize
Source: Front Genet. 2023 Nov 7;14:1282673. doi: 10.3389/fgene.2023.1282673 (PMC10661943; doi:10.3389/fgene.2023.1282673)
Supplement: Supplementary file 2 [file DataSheet1.PDF]

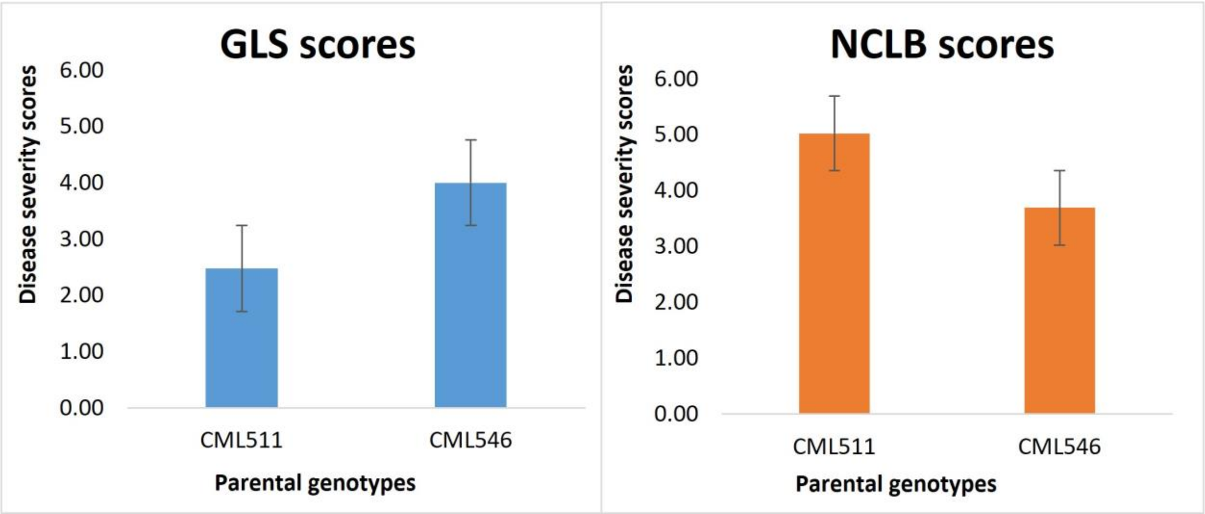

Supplementary Figure S1: GLS and NCLB disease severity scores for the parental genotypes 2 CML511 and CML546 on a scale of 1-9 at the final score across the 3 trial sites.

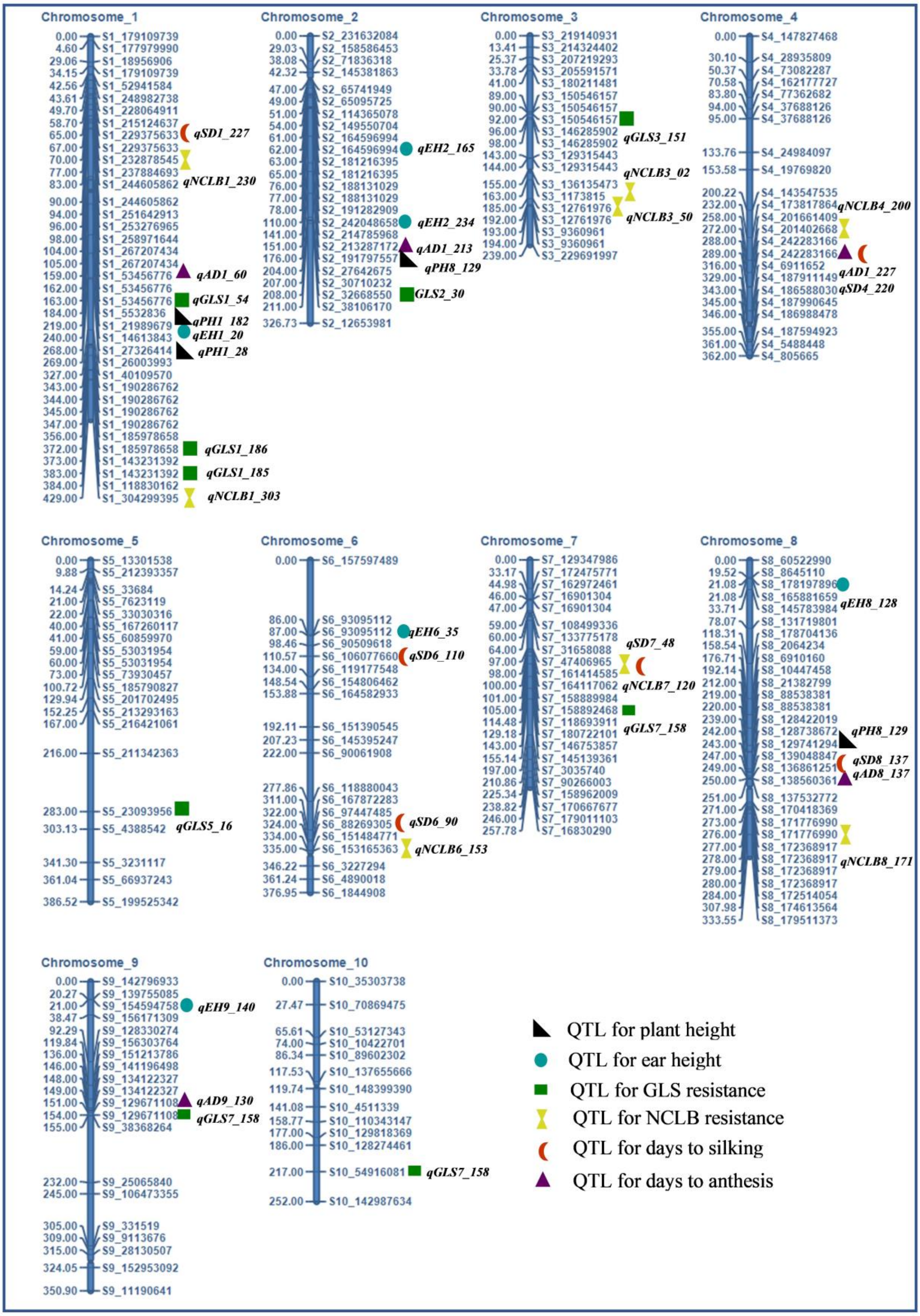

Supplementary Figure S2. The genetic linkage map from the CML511xCML546 DH 5 mapping population shows the distribution of QTLs and QTL positions on the maize 6 chromosomes

combined across the three environments. The solid bars to the right of the chromosome represent the QTL intervals for plant height, ear height, resistance to NCLB, resistance to GLS, days to silking and days to anthesis.

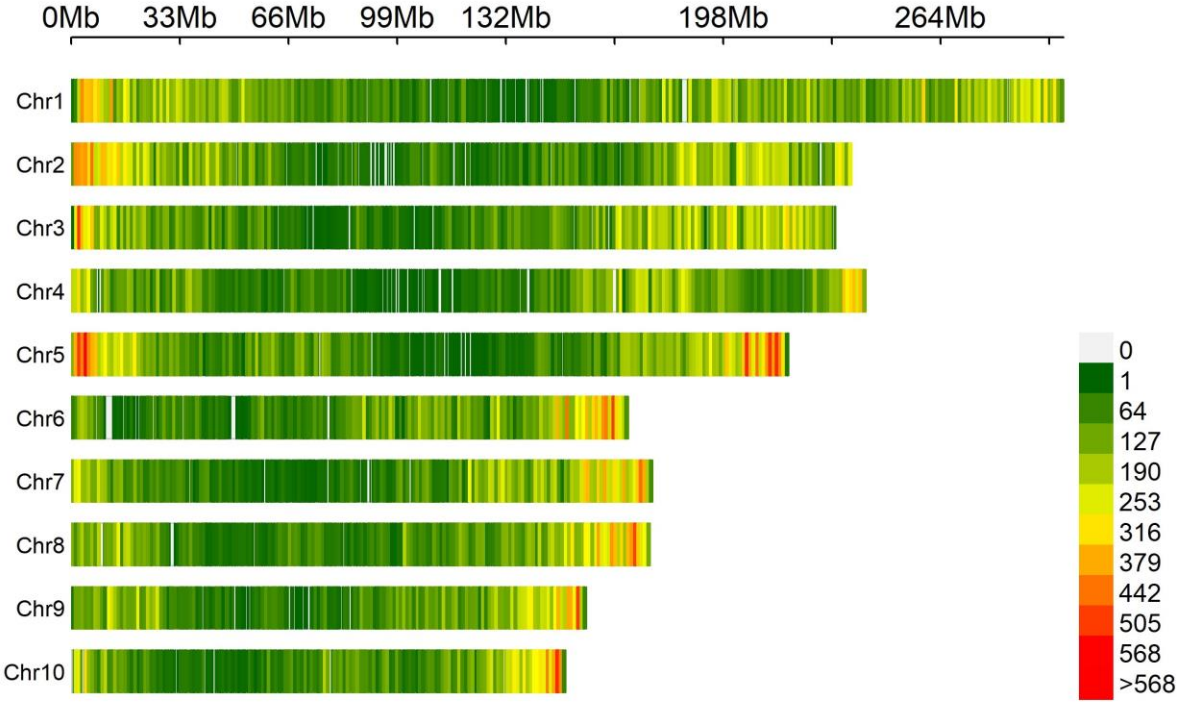

Supplementary Figure S3. Density plot showing the number SNPs within 1 Mb window size from the studied 230,743 markers used for GWAS of the maize association panel. The number of SNPs is displayed on a scale from green to red.

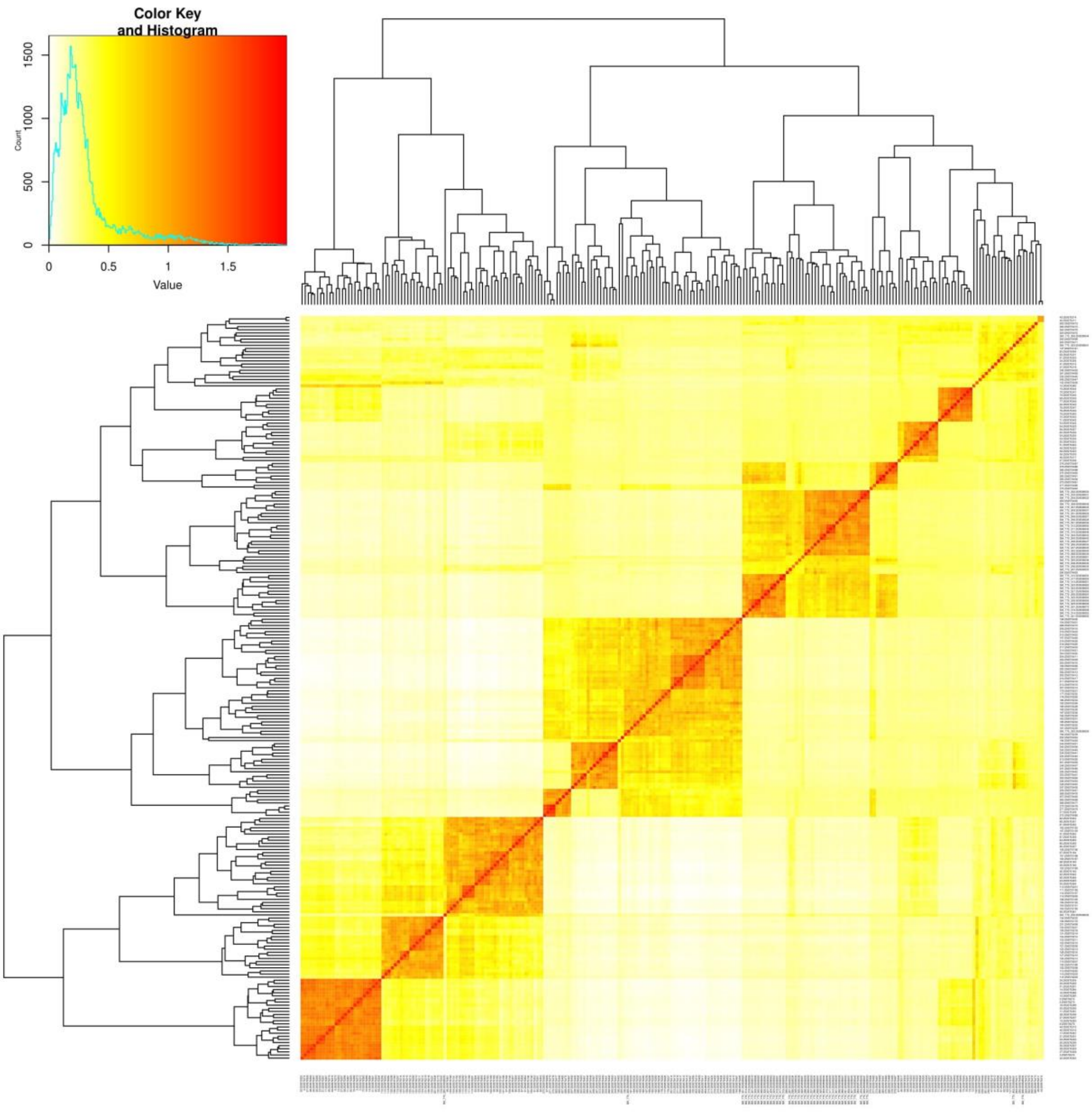

Supplementary Figure S4. Kinship Analysis of the association panel. The heat map shows 17 pairwise kinship matrix based on 230,743 filtered SNPs.

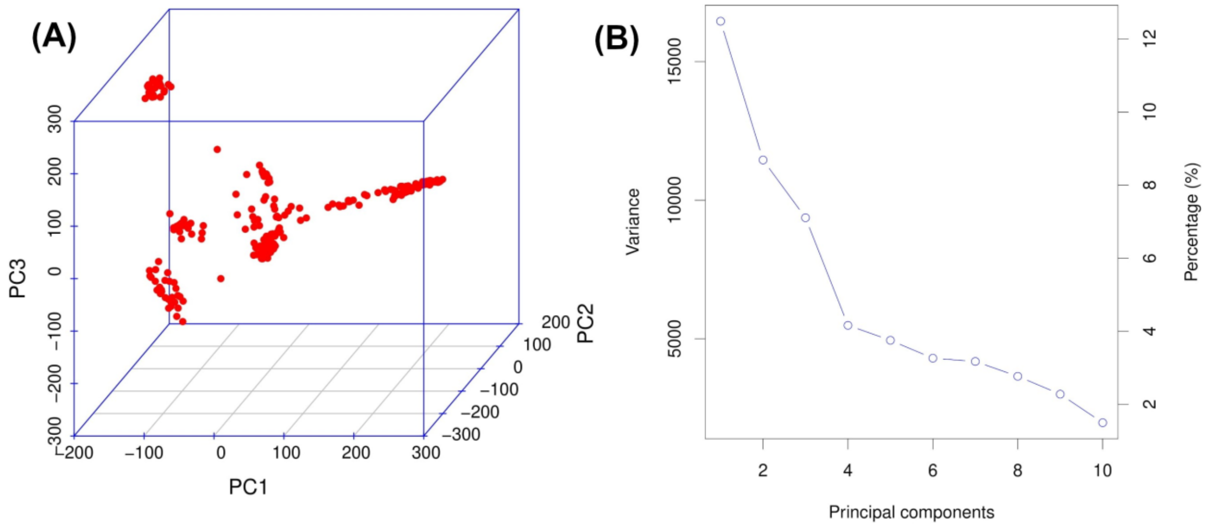

Supplementary Figure S5. (A) Population structure representation based on the first three principal components (PC). (B) Scree plot of the estimated eigen values of each PC. The PCA plots of the association panel were computed using 230,743 SNPs.
